# Reflections on the Prospective Outcomes of Injury Study (POIS; 2006-2023): how population-based research can address Māori outcomes and governance

**DOI:** 10.3389/frma.2023.1212827

**Published:** 2023-09-26

**Authors:** Emma H. Wyeth, Sarah Derrett

**Affiliations:** Ngāi Tahu Māori Health Research Unit, Division of Health Sciences, University of Otago, Dunedin, New Zealand

**Keywords:** Māori, injury outcomes, indigenous research, longitudinal study, research impact

## Abstract

Injury is a leading cause of disability. Twenty years ago, we knew financial costs of injury were high but little was known about the short, medium and long-term outcomes after injury. In 2006, a Pilot Study and engagement with Māori across the country was undertaken to discuss the planned main study to understand how best to design a study that was meaningful and beneficial to Māori and policy-makers. Between 2007–2009, 2,856 injured New Zealanders (including 20% Māori) with an Accident Compensation Corporation (ACC) entitlement claim were recruited to the Prospective Outcomes of Injury Study (POIS). Participants shared detailed information (at 3, 12 and 24 months, and 12-years post-injury) about a broad range of topics including: the injury, socio-demographics, health, health services access, employment and wellbeing. Administrative data about injury-related hospitalisations, the sentinel injury and subsequent injuries were also collected, as well as in-depth qualitative interviews. This paper focuses on the why, how and impacts of POIS, especially in relation to Māori design and approaches, capability and capacity building, and leadership. Focusing on these aspects for Māori within POIS over time has ensured delivery of findings capable of informing and improving outcomes and policy. In particular, POIS has had considerable impact, influencing ACC's research strategy and outcomes' focus, and has provided disability, health, and wellbeing outcomes knowledge previously unavailable, especially for Māori.

## Introduction

Twenty years ago, very little was known about the impact, experiences and outcomes of injured New Zealanders. The estimated prevalence of injury-related disability was high (e.g., a quarter of adults identifying as disabled in New Zealand (NZ) attributed injury as the cause) (Ministry of Health, 2004), and costs for supporting longer-term injury were also high (Accident Compensation Corporation; ACC). In the 2001/02 financial year, ACC spent $1.17billion supporting 150,046 entitlement claims (i.e., claims for injuries likely to result in a week or more off paid work or requiring rehabilitation supports) (Accident Compensation Corporation, 2021), but two-thirds of costs were for people with injuries occurring prior to that year. Despite such high-level information about prevalence and ACC costs, no epidemiological studies of longer-term experiences, costs, and outcomes for injured people had been undertaken in NZ across a range of injury types—and ACC's outcomes information was limited to claim closure and/or return to paid employment. Consequently, in 2004, a funding application was submitted to the Health Research Council of New Zealand (HRC) for developmental research ahead of a potential longitudinal cohort study; this was successful and a pilot study completed in 2006 (Derrett et al., 2010).

This paper focuses on describing the research projects that followed. This includes the research rationale, findings and impact, with a particular emphasis on engagement with Māori and initiatives taken to ensure our research could achieve meaningful and beneficial outcomes, specifically for injured Māori.

## The Prospective Outcomes of Injury Study

**Figure 1:**
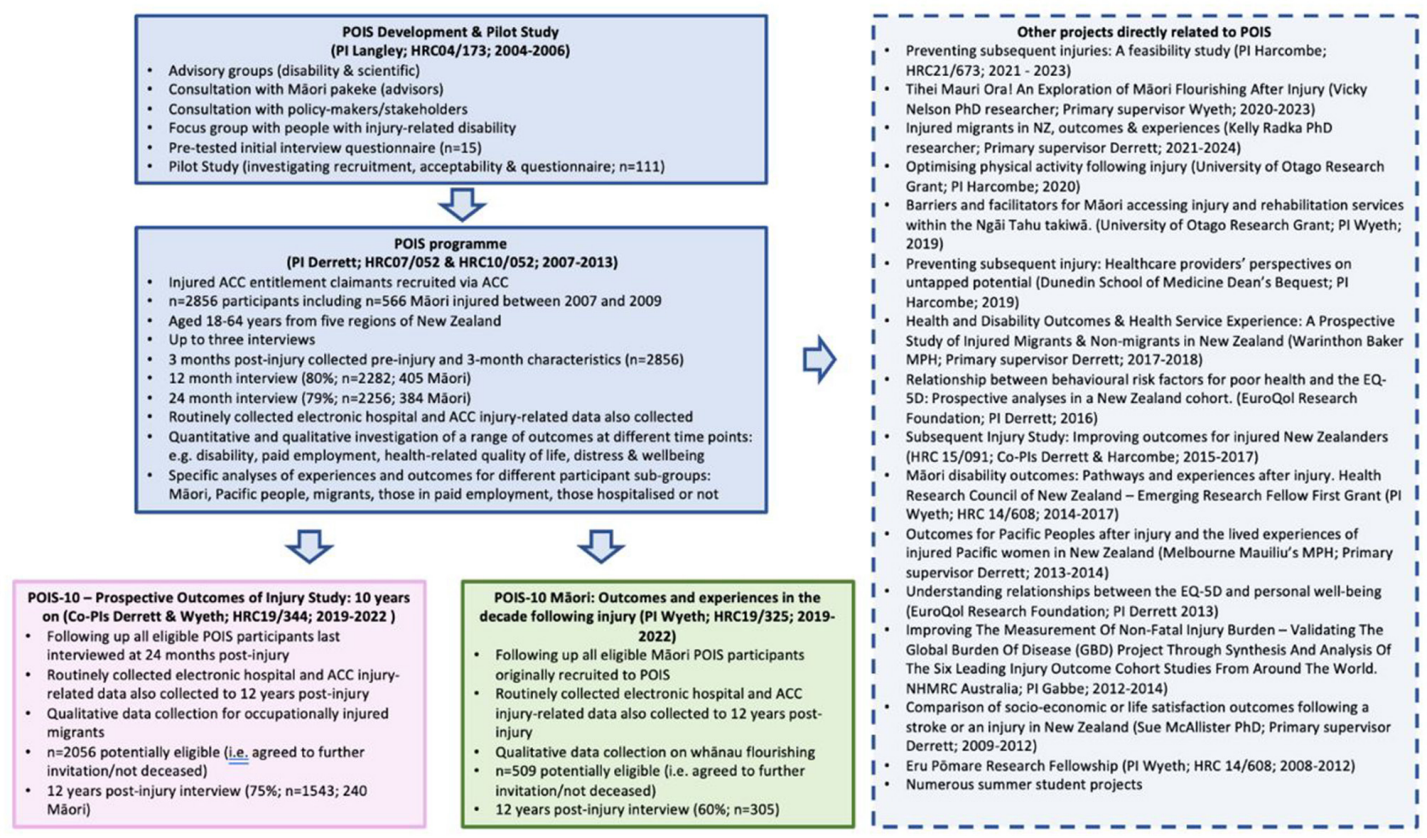
Overview of the Prospective Outcomes of Injury Study (POIS).

The first phase of the Prospective Outcomes of Injury Study (POIS) was funded by the HRC (2010–2013), and co-funded by ACC (2007–2010) (Figure 1). POIS aimed to quantitatively determine the injury, rehabilitation, personal, social and economic factors leading to disability outcomes following injury in NZ, and to qualitatively explore peoples' lived experiences and perceptions of injury-related disability outcomes (Derrett et al., 2011).

The POIS rationale included addressing identified knowledge gaps about injured New Zealanders' outcomes and experiences. Although researchers had qualitatively investigated injury and disability concepts for Māori (Langley and Broughton, 1998; Kingi and Bray, 2000), return to paid work (Crichton et al., 2005), studies tended to be cross-sectional, focused on specific injury types (e.g., fractures or spinal cord injury only) or injury causes (e.g., motor vehicle crashes) (Ameratunga et al., 2006) rather than “all injury.” Internationally, studies had focused on predictors of injury outcomes, but again, these tended to focus on specific injury types (Ottosson et al., 2005), groups (Holbrook et al., 1998, 1999; O'Donnell et al., 2005; Vles et al., 2005), or contained limited measures of predictors or outcomes (Meerding et al., 2004; Vles et al., 2005; Derrett et al., 2011). Additionally, findings from other countries had limited relevance to NZ where ACC is legislatively mandated to prevent, and reduce the severity of, injury. Consequently, POIS sought to understand the short- and medium-term experiences, costs to individuals and whānau, predictors and outcomes for injured New Zealanders for the first time in NZ using relevant measures.

All POIS participants consented to data about their injury and outcomes coming from three sources: (1) interviews with participants held three, 12 and 24 months post-injury, (2) Ministry of Health data about injury-related hospitalisations, and (3) ACC data about participants' injury event, supports and costs (Derrett et al., 2011). The earlier Pilot Study had confirmed the feasibility of inviting potential participants via ACC's entitlement claims register, acceptability of the invitation and interview, appreciation of koha (i.e., voucher) after each interview, and the importance of individual-level information provided by participants remaining confidential to the researchers (and not shared with health providers or ACC) (Derrett et al., 2010).

POIS was a unique study in NZ, where no epidemiological studies had investigated outcomes and experiences for injured New Zealanders with a range of injury causes and types, as well as internationally, where longitudinal studies had been conducted but where participants were typically recruited via trauma units or hospitals. In 2019, we were awarded two further HRC project grants (see Figure 1). One study (POIS-10) was to undertake follow-up of all POIS participants last interviewed 24 months post-injury (Derrett et al., 2021). The other study (POIS-10 Māori) was to undertake follow-up of all Māori POIS participants who had undertaken at least one POIS interview (Wyeth et al., 2021). These two projects included highly-structured follow-up participant interviews (similar to those in POIS) at approximately 12 years post-injury. In addition to these 12-year interviews, both studies also collected data about injury-related hospitalisations to 12 years post-injury and routinely collected data from ACC about the original POIS injury and any subsequent injuries during this follow-up period.

POIS has maintained high follow-up rates at each stage of the longitudinal study, continuing for POIS-10 and POIS-10 Māori. 12-year interviews were undertaken with 75% of eligible POIS-10 participants, and 60% of eligible POIS-10 Māori participants. We believe these longer-term follow-up rates are notable given participants did not have contact with our research team for at least a decade, although we note that the POIS-10 Māori longer-term follow-up rate is lower than for POIS-10. Future analyses will explore some of the reasons for this. However, our earlier analyses to 12 and 24 months post-injury confirmed that Māori participants were more likely than non-Māori to be “lost to follow-up” (Langley et al., 2013). We also found that a range of other factors were independently associated with loss to follow-up, such as inadequate household income and younger age. Much is known about socio-demographic differences between Māori and non-Māori more broadly (Ajwani et al., 2003; Reid and Robson, 2007), and we hypothesise that such differences may contribute to the differential follow-up rates observed. However, the original POIS, and now POIS-10 and POIS-10 Māori studies, comprise the largest longitudinal cohort studies of injured New Zealanders, including injured Māori (*n* = 305 to 12 years post-injury). The following section outlines key initiatives we believe have contributed to this success.

### Designing and undertaking a longitudinal study in New Zealand

In order to ensure meaningful outcomes for Māori, from any study, it is imperative that research be informed and conducted using Māori perspectives, values and methods (Smith, 1999; Cram, 2001; Simmonds et al., 2008). Many efforts were undertaken to ensure that POIS, and related studies, were developed in this way (Wyeth et al., 2010, 2021). When the idea of a longitudinal injury study was first conceived, the importance of ensuring Māori leadership and involvement was recognised by the Principal Investigator (PI Langley) of the developmental study. As a result, a senior Māori academic joined the team at that early stage. Additional efforts were undertaken over subsequent years to ensure that a longitudinal injury study was able to contribute significantly to the limited knowledge available about Māori injury outcomes (Wyeth et al., 2010).

A crucial component of POIS has been that more Māori academics have joined the team over time as named investigators, associate investigators, advisors and post-graduate students. A continuation of this explicit contribution to the Māori health research workforce, and leadership especially, is seen in POIS-10 (with EW as Co-PI with SD), and in POIS-10 Māori (with EW as PI). Additionally, the aim was to recruit sufficient Māori participants to enable stand-alone analyses, with a goal of 20% of the cohort being Māori. POIS recruitment continued until this was achieved; with 566 injured Māori forming the baseline Māori POIS cohort. Questionnaires were translated into te reo Māori (which required permission to lead the translation of validated international measures included within POIS questionnaires), Māori interviewers were employed and were specifically available for Māori participants, and for requested face-to face interviews. We believe such actions have contributed to our recruitment and follow-up success.

Further, we have data sovereignty guidelines. All project investigators and associates are required to sign an agreement that includes specific sections on principles relating to requests to use Māori data and conduct analyses according to ethnicity. These guiding principles have been developed over the years and explicitly state that, as part of our Treaty of Waitangi commitments:

- We aim to prevent a deficit model of analysis and to ensure meaningful research outcomes for Māori.
- When analyses include ethnicity, particular care must be taken in relation to the clear unambiguous presentation and interpretation of results (e.g., where it is hypothesis-generating this is clearly indicated).
- Particular attention should be paid to the language and framing of results in relation to ethnicity to avoid potential deficit and/or negative statements, including by others outside the team.

Investigators are asked to provide the PIs with a rationale for proposed analyses by ethnicity or use of Māori data for consideration and discussion. Before approval, potential risks and benefits are considered by the PIs and other Māori investigators. Considerations include whether Māori investigators should be involved in the proposed work and publications as co-authors. If co-authorship is deemed to not be required, full draughts of analyses and interpretations must be provided to Māori investigators for comment and/or suggested amendments.

Twelve years later, with the development of POIS-10 Māori, kaupapa Māori research principles and models of Māori health explicitly underpin the study, and guide questionnaire development, analyses and interpretation. The vast majority of the research team is Māori from a range of career stages, including a PhD student, and six of seven advisors are Māori. We have again translated the Study Information Sheet into te reo Māori, made our study documents more visually appealing to Māori, and employed Māori interviewers. We have had continued success with participation in our follow-up interviews. Some participants who were not contactable for a 24-month POIS interview, some of whom had previously only completed the first POIS interview 3 months post-injury, have since been located and re-engaged for POIS-10 Māori and completed a 12-year interview. This alone is testament that what we have done, and are doing, is supporting Māori engagement and participation in longitudinal research. Despite no interviewer contact during this lengthy period, participants have remembered their previous interview(s), appreciated koha provided after each interview, and received regular study newsletters all helping to maintain connexions between participants and the research team.

POIS-10 also has a strong relationship with, and is hosted by, the Ngāi Tahu Māori Health Research Unit (EW is the Co-Director). POIS-10 Māori was also used as a starting point to guide the development of the POIS-10 questionnaire. Specifically, the research team first identified what we wanted to ask Māori participants and then considered whether such questions should be asked of all participants. As a result, there are only three additional questions asked only of Māori participants (e.g., relating to being an owner or beneficiary of Māori land). Likewise, and after discussions with ACC, we are currently developing a potential intervention intended to help identify injured people who may benefit from additional/earlier ACC support. Interventions and risk models developed in Europe, or with non-Indigenous populations, have been found to be less effective for other groups, sometimes exacerbating inequities (Wang and Hoy, 2005; Kirmayer and Brass, 2016; Tran-Duy et al., 2020). Therefore, we are developing a tool to support interventions from a Māori perspective and with a principled approach that what is good, or works well, for Māori, should work well for others.

### Impact of research

The design of POIS, and now POIS-10 and POIS-10 Māori, and the unique linking of self-reported interview data about experiences and outcomes with large injury-related administrative datasets means our longitudinal study has been able to address national and international knowledge gaps. Our study has identified specific injury, rehabilitation, personal, vocational and social factors leading to a range of outcomes up to 24 months (Derrett et al., 2011), and is now possible to 12 years (Derrett et al., 2021; Wyeth et al., 2021), following a significant injury event. Results for the whole cohort, and specifically for Māori (Maclennan et al., 2013, 2014; Wyeth et al., 2013, 2017, 2018; Wyeth E. et al., 2019), have revealed key predictors of disability (Lilley et al., 2012, 2017; Derrett et al., 2013; Langley et al., 2014; Wyeth et al., 2018; Wyeth E. H. et al., 2019), and other health outcomes [e.g., subsequent injury events (Harcombe et al., 2014, 2017; Wyeth E. et al., 2019), health-related quality of life (Wilson et al., 2017), physical functioning (Harcombe et al., 2015), wellbeing (Wyeth et al., 2013), and distress (Ameratunga et al., 2021; Richardson et al., 2021)], using validated measures.

POIS, and related studies, have now been running across six national election cycles. Seemingly related to major electoral changes, ACC (a Crown entity) has had considerable changes in management, structure and personnel. Some changes have resulted in periodic losses including Māori leadership, institutional knowledge, and even the loss of certain publicly available useful data about ACC's performance. Despite political twists and turns, and multiple restructures and personnel changes, ACC has been and remains a key supporter of POIS, POIS-10 and POIS-10 Māori.

Sometimes it can be difficult as researchers to be confident about the reach or uptake of findings. However, POIS has informed ACC's long-term priorities and its focus on life-course perspectives, outcomes and hauora or wellbeing (personal communication, 2019 letter from ACC to SD and EW). Additionally, building on POIS, our study of subsequent injuries (Derrett et al., 2017; Harcombe et al., 2017), has informed ACC's re-injury and subsequent injury prevention work-stream. ACC has also told us that the POIS research team's trusted relationships with participants, and our methods of data collection, have led to (de-identified) knowledge about injured New Zealanders' participation, disability, health-related quality of life and wellbeing outcomes important for ACC. Key to the impact from our longitudinal study, and translation of POIS findings into practise, has been the constructive relationships between our research team and ACC.

## Discussion

In developing a longitudinal study of injured New Zealanders, with a clear goal of contributing to understanding and improving injury outcomes for injured Māori specifically, we have had many other successes. We have published over 60 peer-reviewed articles, many of which have been cited widely. We have presented at international, national and local conferences, symposia and hui to a wide range of audiences and disciplines including injury, Indigenous health, disability, rehabilitation, quality of life, wellbeing, health services and systems. We have worked with and supported at least 17 named investigators, 25 advisors, 10 associate investigators, 11 post-graduate or Summer students, and four post-doctoral fellows – a significant contribution to capacity building within the health research workforce over 20 years. POIS has also provided academic career progression opportunities. For example, EW originally joined POIS as an investigator on a HRC Māori Post-doctoral Fellowship, then progressed to PI of a Māori-specific project directly related to POIS (see Figure 1) and is now PI of POIS-10 Māori and Co-PI of POIS-10. Such career building opportunities are enhanced when Māori students and early career academics can see pathways to and beyond post-graduate studies. Amongst our large collaborative group, a number of people on our research and advisory teams have remained constant which has been critical for maintaining in-depth knowledge about the study and its sustainability.

While not all funding applications have been successful, we have received significant ongoing funding from the HRC enabling the longevity of POIS, for which we are grateful. The contestable nature of research funding is important, and helps maintain high quality excellent research, but this creates an increased risk for the continuation of longitudinal studies which add significantly important richness to our understanding of health and wellbeing in NZ.

Our data collection methods, where interviewers are home-based and interviews are conducted via telephone and responses are entered directly into a secure online database, has also worked well. Additionally, 10 days after starting data collection for POIS-10 and POIS-10 Māori in March 2020, NZ went into a national “lockdown” and Alert Level 4 due to the COVID-19 pandemic. We paused data collection while the research team, interviewers and potential participants made necessary arrangements. This enabled us to add additional COVID-19-related questions to capture people's COVID-19 experiences and impacts, but it also meant that we were able to resume data collection within a relatively short timeframe and continue during “lockdown” unlike some other research projects.

The constant independence and impartiality from government entities, and our very firm commitment to not sharing identifiable information with government agencies or healthcare services has been important. We made this very clear to participants at every interview, which we hypothesise has further contributed to our high follow-up rates. Despite this, it is important that we continually engage with key organisations and agencies (e.g., ACC) to ensure translation and uptake of findings, and ensure new knowledge gaps are identified for future investigation. Despite the challenges resulting from political and institutional changes, longitudinal studies such as ours, have opportunities to build and foster longer-term researcher-stakeholder relationships to facilitate regular knowledge translation in a way that may not be available to shorter-term research projects.

With a number of successes, and the benefit of hindsight, we have reflected on what we might have done differently if embarking on establishing a new longitudinal study now. We would have set out to recruit participants to ensure equal explanatory power (i.e., equal numbers of injured Māori and non-Māori) (Te Ropu Rangahau Hauora a Eru Pomare., 2002), and would have employed more interviewers proficient in a greater number of languages.

It is noteworthy that NZ has experienced considerable social change throughout the 20 year history of POIS. Additionally, the NZ research landscape and context is now very different. Even though we have been careful and intentional in the design and development of POIS to ensure a study that is relevant and able to contribute to knowledge gaps specifically for Māori, recent changes to the health research sector, e.g., updated requirements and expectations of researchers seeking HRC funding via the Māori Health Advancement Guidelines (Health Research Council of New Zealand., 2019) have also supported the continued enhancements we have made to POIS over the last 20 years. We believe that such changes will also result in a greater number of studies contributing more significantly to Māori health advancement and addressing the considerable and persistent inequities that Māori experience. This is a future we very much look forward to.

## Data availability statement

The data analysed in this study is subject to the following licences/restrictions: Descriptive data are reported in the paper. POIS-10 data use is restricted due to the Study Participant Information Sheet and Consent Form, which explicitly state collected data will not be shared publicly. Qualified researchers, and postgraduate students, may request to collaborate with the POIS-10 research team by contacting the corresponding author. Requests to access these datasets should be directed to EW (emma.wyeth@otago.ac.nz) and SD (sarah.derrett@otago.ac.nz).

## Ethics statement

The studies involving human participants were reviewed and approved by Southern Health and Disability Ethics Committee (MEC/07/07/093/AM07). Written informed consent was not provided because all participants received detailed Study Information Sheets and provided full oral consent prior to participating in POIS, POIS-10 and POIS-10 Māori, and received a hard copy of the Consent Form for their records.

## Author contributions

All authors listed have made a substantial, direct, and intellectual contribution to the work and approved it for publication.
